# Evaluation of Prognosis and Risk Factors of Fulminant Myocarditis Complicated with Malignant Arrhythmia

**DOI:** 10.3390/jcdd13010014

**Published:** 2025-12-24

**Authors:** Yanan Wang, Jialin Zang, Guangling Li, Zeping Li, Luyun Wang, Jiangang Jiang

**Affiliations:** Department of Cardiology, Tongji Hospital, Tongji Medical College, Huazhong University of Science and Technology, Jiefang Avenue 1095, Wuhan 430030, China; 15036159279@163.com (Y.W.); m202476601@hust.edu.cn (J.Z.); d202382293@hust.edu.cn (G.L.); d202482521@hust.edu.cn (Z.L.)

**Keywords:** fulminant myocarditis, malignant arrhythmia, risk factor, prognosis

## Abstract

(1) Background: Malignant arrhythmia complicating fulminant myocarditis is associated with high in-hospital mortality, but evidence regarding its long-term prognosis and specific risk factors is limited. (2) Methods: This single-center retrospective cohort study (2016–2025) analyzed 241 consecutive fulminant myocarditis patients, stratified by malignant arrhythmia status (*n* = 58 vs. 183). The malignant arrhythmia group was further subclassified into malignant tachyarrhythmia (*n* = 22) and bradyarrhythmia (*n* = 36). Endpoints included major adverse cardiovascular events (MACE), cardiac dysfunction, and structural abnormalities. (3) Results: At 3-month follow-up, malignant arrhythmia patients had a significantly higher incidence of MACE compared to non-malignant arrhythmia patients (15.5% vs. 4.9%, *p* = 0.008), but no significant differences were found in cardiac dysfunction or structural abnormalities. Multivariate analysis identified low triglyceride level as an independent risk factor for in-hospital malignant tachyarrhythmia. For in-hospital malignant bradyarrhythmia, independent risk factors were delayed, such as intrinsicoid deflection, low diastolic blood pressure, bradycardia, and an elevated E/Em ratio, with the predictive model showing high discriminatory power. (4) Conclusions: Malignant arrhythmia is an independent predictor of adverse short-term, but not long-term, prognosis in fulminant myocarditis patients, with distinct risk factor profiles identified for malignant tachyarrhythmia and malignant bradyarrhythmia subtypes.

## 1. Introduction

Myocarditis refers to the inflammation of the myocardium, which can lead to tissue degeneration or necrosis [1]. Acute myocarditis, which accounts for approximately 65% of all cases of myocarditis, is mainly due to viral infections, although noninfectious causes may also play a role. The clinical presentation of acute myocarditis is variable and may include febrile illness, mild chest pain, arrhythmias, heart failure, cardiogenic shock, or sudden death [2]. Fulminant myocarditis represents the most severe form, characterized by rapid onset and progression. Patients can develop severe heart failure, hypotension/cardiogenic shock, and malignant arrhythmias within a very short timeframe, sometimes leading to sudden death [3,4,5]. Arrhythmias occur in approximately 20% to 30% of FM patients [3]. Severe arrhythmias can induce or exacerbate hemodynamic instability, posing a significant threat to patient survival [4]. Cardiogenic shock and electrical instability, such as recurrent ventricular tachycardia or advanced atrioventricular block, are key risk factors for adverse outcomes in myocarditis [1]. Malignant arrhythmias serve as a critical warning sign for the progression from acute myocarditis to FM, indicating a rapid deterioration towards a critical state [6]. Inflammation, abnormal myocardial energy metabolism, and direct myocardial injury (apoptosis and necrosis) play an important role in the development of arrhythmia [7]. Currently, research regarding the impact of MA on the prognosis of patients with FM remains scarce. Additionally, there are no studies exploring which patients with FM are predisposed to developing MA. Our study aimed to examine the association between MA and prognosis in patients with FM and to analyze risk factors influencing the occurrence of MA in this population.

## 2. Material and Methods

### 2.1. Study Design and Population

This retrospective cohort study enrolled 241 patients with FM hospitalized in the Cardiology Department of Tongji Hospital affiliated to Tongji Medical College of Huazhong University of Science and Technology from April 2016 to July 2025.

The diagnosis of FM includes both clinical and pathological diagnoses. Current pathological diagnosis mainly relies on Endomyocardial Biopsy (EMB) to obtain myocardial tissue [8]. Because of the limitations, such as its reliance on physician experience and complications [9], only parts of the FM receive EMB. Thus, the diagnosis of FM in this study is also based on clinical manifestations and cardiac magnetic resonance imaging (CMRI). According to the Chinese expert consensus statement [3], the following requirements must be satisfied: (1) prodromal symptoms of upper respiratory or gastrointestinal viral infections, particularly fever, fatigue, and poor appetite; (2) rapid development of hemodynamic compromise requiring inotropic drugs or mechanical life support (MLS); (3) elevated serum levels of myocardial necrosis biomarkers; (4) significant ECG changes (low voltage, extensive lead ST segment and T wave changes and conduction block, etc.) or echocardiography abnormities (such as diffuse wall hypokinesia, markedly decreased left ventricular ejection fraction); and (5) exclusion of other cardiac diseases, including valvular disorders, acute coronary syndrome, acute ischemic cardiomyopathy, or illnesses showing comparable clinical symptoms [10,11,12]. Exclusion criteria: (1) refusal to follow-up; (2) age < 14 years old; (3) patients with other preexisting conditions that may lead to changes in cardiac structure and function, including malignant tumor, myocardial infarction, etc. The following arrhythmias were identified on any of the electrocardiograms, holter, or electrocardiographic monitoring performed during hospitalization. Every patient remained under comprehensive surveillance during hospitalization. MA refers to the types of arrhythmias such as cardiac arrest, ventricular fibrillation, ventricular flutter, sustained ventricular tachycardia (lasting >30 s or requiring termination due to hemodynamic instability) [13], and third-degree atrioventricular block and second-degree atrioventricular block, Mobitz type II. They were further divided into two subgroups according to tachycardia or bradycardia. Malignant tachycardia (MT) consists of cardiac arrest after ventricular fibrillation (VF), ventricular fibrillation, ventricular flutter (VT) and sustained ventricular beats, while malignant bradycardia (MB) includes cardiac arrest after third-degree atrioventricular block, and third-degree atrioventricular block and second-degree atrioventricular block, Mobitz type II.

### 2.2. Study Endpoint and Patient Follow-Up

The primary endpoint was MACE [14], composed of cardiac death, cardiogenic shock, and heart failure:

1. Cardiac death: death attributable to cardiac causes, including fatal myocardial infarction, heart failure, or arrhythmia, after excluding non-cardiac causes.
2. Cardiogenic shock: a clinical condition characterized by persistent hypotension and tissue hypoperfusion due to cardiac dysfunction.
3. Heart transplantation.
4. Malignant arrhythmia: documented cardiac arrest, ventricular fibrillation, ventricular flutter, sustained ventricular tachycardia (lasting >30 s or requiring termination due to hemodynamic instability), third-degree atrioventricular block and second-degree atrioventricular block, Mobitz type II.
5. Recurrent myocarditis: avnew episode of acute myocarditis confirmed by reappearance of symptoms, elevated cardiac biomarkers (troponin), and supportive cardiac imaging (CMR) or endomyocardial biopsy findings after the initial episode had resolved.
6. Hospitalization for heart failure: an unplanned hospital admission primarily for the management of worsening heart failure, requiring intravenous diuretic or inotropic therapy.

Secondary outcome measures of the study focused on the functional and structural abnormalities of the left heart [15]. Functional abnormality: Left ventricular ejection fraction (LVEF) < 50% as measured by echocardiography. Structural abnormality: Sex-specific chamber enlargement defined as follows: LV end-diastolic diameter > 5.5 cm or left atrial diameter > 4.0 cm (men) as measured by echocardiography; LV end-diastolic diameter > 5.0 cm or left atrial diameter > 3.5 cm (women) as measured by echocardiography. Patients underwent protocolized follow-up at 1, 3, and 6 months and biannually thereafter, with proactive tracing ensuring the timely completion of follow-up assessments.

### 2.3. Data Collection

Standardized data extraction from electronic records captured demographics, admission vitals, treatment modalities, and outcomes. Laboratory analysis included cardiac biomarkers (troponin, NT-proBNP), inflammatory markers, and routine biochemistry. Electrocardiogram (ECG) and Holter parameters primarily comprised measures related to heart rate, rhythm, wave intervals, voltage, axis, and heart rate variability (HRV). All patients underwent standardized echocardiography to evaluate LVEF, left ventricular end-diastolic diameter (LVEDd), left atrial diameter (LAD), early to late diastolic mitral inflow velocity ratio (E/A), global longitudinal strain (GLS), and Early Diastolic Mitral Inflow Velocity to Early Diastolic Mitral Annular Velocity Ratio (E/Em). Functional and structured clinical follow-ups were conducted at 1, 3, and 6 months post-discharge, followed by biannual assessments as part of routine clinical care. Follow-up assessed survival status and standardized echocardiographic parameters. Patients who did not attend scheduled visits were contacted by phone; those lost to follow-up were censored at their last known evaluation date. For the analysis of left ventricular ejection fraction (LVEF) trajectories, missing echocardiographic data were handled using the last observation carried forward (LOCF) method under the assumption that data were missing at random.

### 2.4. Statistical Analysis

Continuous variables ware presented as mean ± standard deviation (SD) for normally distributed data or median (interquartile range; IQR) [reported as M (Q1, Q3)] for non-normally distributed data. Intergroup comparisons for continuous variables were performed using the independent samples *t*-test for normally distributed data and the Mann Whitney U test (rank sum test) for non-normally distributed data. Categorical variables were summarized as frequencies (percentages) and compared between groups using the χ^2^ test or Fisher’s exact test, as appropriate. Cumulative incidence rates of secondary outcomes during long-term follow-up were estimated using Kaplan Meier analysis, with between-group comparisons assessed by the log-rank test. Logistic regression was used to analyze the risk factors of the occurrence of MA in FM during hospitalization. Univariate analysis identified significant variables (*p* < 0.05), which we then entered into the multivariate logistic regression model after confirming the absence of multicollinearity. Considering that troponin is associated with the overall severity of fulminant myocarditis, it was included in the multivariate logistic regression analysis regardless of whether it reached statistical significance. The predictive value of the risk factors for MA during hospitalization was assessed by ROC curve analysis. A two-sided *p*-value < 0.05 was considered statistically significant for all analyses. SPSS 25.0 and R 4.5.0were used for statistical analyses.

## 3. Result

### 3.1. Malignant Arrhythmias Increase Short-Term but Not Long-Term Cardiovascular Risk in Fulminant Myocarditis

**Figure 1:**
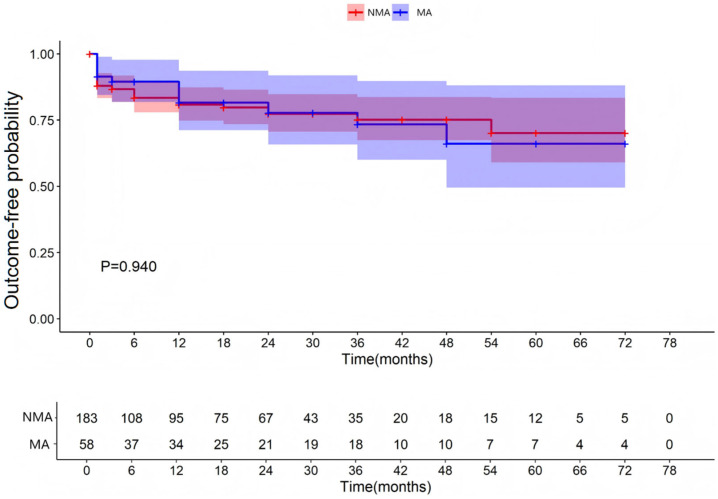
Occurrence of functional abnormalities of the left heart. NMA indicates non-malignant arrhythmia group and Ma indicates malignant arrhythmia group.

**Figure 2:**
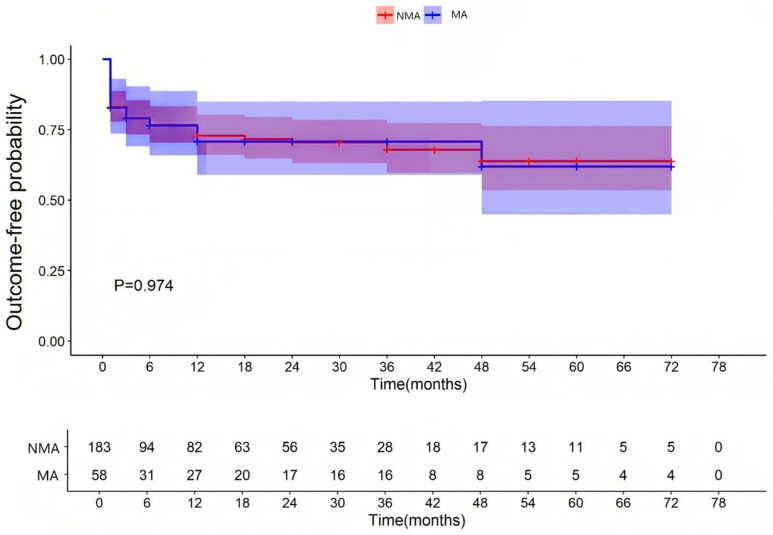
Occurrence of structural abnormalities of the left heart. NMA indicates non-malignant arrhythmia group and MA indicates malignant arrhythmia group.

All patients were followed for up to 12 months after hospital discharge. The median follow-up duration was 12 months (interquartile range [IQR]: 3–36). A total of 183 patients with fulminant myocarditis were allocated to the MA group (with malignant arrhythmias) and 58 patients to the NMA group (without malignant arrhythmias). These patients were followed up after discharge. As shown in Table 1 and Supplementary Materials Table S1, FM patients with MA group had a significantly higher 3-month post-discharge MACE incidence (15.5% vs. 4.9%, χ^2^ = 5.708, *p* = 0.017), but a similar incidence after 3 months (3.4% vs. 2.2%, χ^2^ = 0.003, *p* = 0.957). For functional abnormalities, comparable event-free survival was observed through 78 months (*p* = 0.940; Figure 1); for structural abnormalities, equivalent remodeling risk was maintained through 78 months (*p* = 0.974; Figure 2).

Because significant differences were observed in patients with bradyarrhythmia and tachyarrhythmia, particularly in ECG parameters, the MA group was subdivided into malignant bradyarrhythmia (MB) and malignant tachyarrhythmia (MT) subgroups.

### 3.2. The Subgroup Analysis Between MT and NMA

#### 3.2.1. General Characteristics

MT group (*n* = 22) had a significantly lower proportion of males (22.7% vs. 53.6%, *p* = 0.006) and a longer length of hospital stay (*p* = 0.024). Additionally, systolic and diastolic blood pressure at admission was significantly lower in the MT group (all *p* < 0.05). Other indicators had no statistical difference.

#### 3.2.2. Laboratory Examinations

The MT group had significantly higher baseline levels of cardiac troponin I (cTnI) (*p* = 0.023) and interleukin-10 (IL-10) levels (*p* = 0.037), but significantly lower C-reactive protein (CRP) [11.5 (4.2, 26.4) vs. 32.6 (9.4, 79.0), *p* = 0.021] and erythrocyte sedimentation rate (ESR) [7 (3, 17) vs. 12 (5,24), *p* = 0.033] than the NMA group (Table 2 and Table S3).

#### 3.2.3. Electrocardiogram, Dynamic Electrocardiogram, and Echocardiographic Findings

Analysis of ECG revealed the longer Tpeak-to-Tend (TpTe) interval in the MT group [97 (78, 150) vs. 81 (73, 117), *p* = 0.030)], significantly higher root mean square of successive differences (rMSSD) [43 (29, 79) vs. 28 (18, 56), *p* = 0.031], and lower P wave width [82 (60, 93) vs. 92 (76, 100), *p* = 0.035]. Echocardiographic assessment showed that left ventricular ejection fraction (LVEF) at admission was a little lower in the MT group but did not reach statistical significance [33 (25, 44) vs. 40 (28, 52), *p* = 0.120] and the absolute value of global longitudinal strain (GLS) was significantly lower (−7.3 ± 4.3 vs. −9.7 ± 5.0, *p* = 0.027), which indicates worse function in the MT group compared to the NMA group (Table S3).

#### 3.2.4. Treatment

The proportion of patients with extracorporeal membrane oxygenation (ECMO), vasoactive drugs (dopamine and metaraminol), pacemaker, continuous renal replacement therapy (CRRT), invasive mechanical ventilation (IMV), and CPR/defibrillation were higher than that in the NMA group (all *p* < 0.05), and the initial dose of glucocorticoids was also higher (Table 3). This is because patients with MT often have more severe hemodynamic disturbances. Because the use of CPR/Defibrillation often took place after MT, it was not incorporated into subsequent analysis.

#### 3.2.5. Risk Factors for MT in Patients with FM

**Figure 3:**
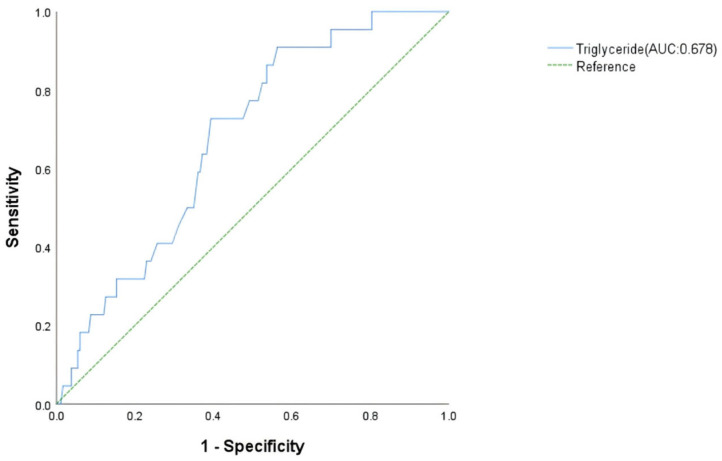
Receiver operating characteristic analysis of malignant tachyarrhythmia risk factors in fulminant myocarditis patients.

Following screening by univariate analysis (Table 4) and assessment for multicollinearity (VIF < 5) (Table 5), a multivariate logistic regression model was constructed. The multivariate logistic regression analysis showed that lower triglyceride was an independent risk factor for MT in patients with FM. The odd ratio for MT was 0.240 (*p* = 0.047, 95% CI: 0.058–0.984) per 1 mmol/L increase in triglyceride (Table 6). The AUC was 0.678 (*p* = 0.050, 95% CI: 0.577–0.779) for triglyceride (Figure 3).

### 3.3. The Subgroup Analysis Between MB and NMA

#### 3.3.1. General Characteristics

Compared to the NMA group, MB patients were older [47 (27, 57) vs. 36 (22, 49), *p* = 0.026]. The diastolic blood pressure was lower in the MB group (58 ± 11 vs. 65 ± 13, *p* = 0.004) (Supplementary Materials Table S4). The sex ratio and length of hospital stay were similar.

#### 3.3.2. Laboratory Examinations

Alanine aminotransferase (ALT) was higher in the MB group than in the NMA group [87 (32, 257) vs. 49 (28, 93), *p* = 0.039]. None of inflammatory markers showed difference between groups (Supplementary Materials Tables S4 and S5).

#### 3.3.3. Electrocardiogram, Dynamic Electrocardiogram, and Echocardiographic Findings

ECG analysis revealed many differences, among which heart rate [81 (60, 98) vs. 93 (79, 117), *p* = 0.001], P wave width [80 (67, 94) vs. 92 (80, 100), *p* = 0.002], QRS wave width [130 (98, 153) vs. 96 (84, 116), *p* = 0.000], PR interval [166 (149, 192) vs. 150 (130, 169), *p* = 0.004], RV5 + SV1 [0.76 (0.24, 1.21) vs. 0.94 (0.48, 1.59), *p* = 0.012], Frontal QRS-T angle [−36.5 (−118.4, 55.6) vs. 22.4 (−36.6, 60.5), *p* = 0.011], delayed intrinsicoid deflection [18 (50.0) vs. 24 (13.1), *p* = 0.000], delayed QRS transition [18 (50.0) vs. 24 (13.1), *p* = 0.006], maximum heart rate (105 ± 19 vs. 115 ± 18, *p* = 0.005), and triangulation index [14.3 (5.8, 23.4) vs. 17.5 (11.5, 25.7), *p* = 0.043] were more significant. Echocardiographic assessment showed that only E/Em [19 (12, 27) vs. 13 (9, 19), *p* = 0.000] had a statistically significant difference between the two groups (Supplementary Table S4).

#### 3.3.4. Treatment

The MB group required more frequent CPR/Defibrillation and pacemaker implantation, but lower ACEI/ARB and beta blockers usage (all *p* < 0.05; Supplementary Table S6). These treatment differences primarily reflect MB-specific clinical management needs rather than possessing independent predictive value.

#### 3.3.5. Risk Factors for MB in Patients with FM

**Figure 4:**
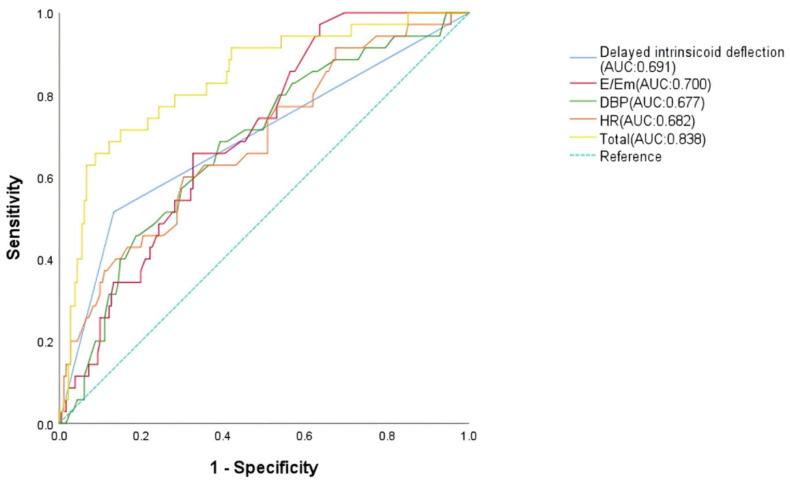
Receiver operating characteristic analysis of malignant bradyarrhythmia risk factors in malignant arrhythmia patients.

Following screening by univariate analysis (Supplementary Table S7) and assessment for multicollinearity (VIF < 5) (Supplementary Table S8), a multivariate logistic regression model was constructed. The multivariate logistic regression analysis showed that HR or DBP decrease, delayed intrinsicoid deflection, and E/Em increase were independent risk factors for MB in patients with FM during hospitalization; the odd ratio of MB patients with quick HR was 0.784 (*p* = 0.025, 95% CI: 0.639–0.962), with low DBP was 0.651 (*p* = 0.025, 95% CI: 0.446–0.948), with delayed intrinsicoid deflection was 3.113 (*p* = 0.046, 95% CI: 1.020–9.498), and with high E/Em was 1.065 (*p* = 0.010, 95% CI: 1.015–1.116) (Supplementary Table S9). The predictive value of HR, DBP, delayed intrinsicoid deflection, E/Em, and their combination for MB was analyzed using ROC. The AUC, respectively, were 0.682 (SE = 0.050, *p* = 0.001, 95% CI: 0.583–0.780), 0.677 (SE = 0.049, *p* = 0.001, 95% CI: 0.581–0.772), 0.691 (SE = 0.054, *p* = 0.000, 95% CI: 0.584–0.797), 0.700 (SE = 0.042, *p* = 0.000, 95% CI: 0.584–0.797), and 0.838 (SE = 0.038, *p* = 0.000, 95% CI: 0.763–0.913) (Figure 4).

## 4. Discussion

This retrospective study of 241 FM patients found that MA significantly impacted short-term prognosis, but had a minimal effect on long-term prognosis, such as changes in cardiac structure and function. While MA patients faced high acute-phase mortality, those surviving this critical period experienced favorable long-term prognoses, with MACE predominantly occurring within the acute window [16]. Given that chronic myocarditis typically requires ≥3 months of persistent inflammation for diagnosis [17], we used this threshold to analyze MACE timing. Long-term follow-up revealed no increased cardiac structural and functional abnormalities in MA patients, indicating that MA represent transient electrical instability during acute inflammatory phase. Following inflammatory resolution, electrophysiological homeostasis is restored without inducing persistent myocardial injury [18]. Conversely, chronic-phase ventricular dysfunction primarily arises from fibrotic remodeling secondary to myocarditis [19], rather than arrhythmic burden. This dichotomy underscores that arrhythmias in FM serve as epiphenomena of acute inflammation, whereas fibrosis drives chronic cardiomyopathy.

The MT group demonstrated a higher proportion of females. This gender disparity aligns with evidence indicating poorer prognosis in female myocarditis patients [20]. Experimental studies suggest that sex hormones contribute mechanistically: testosterone may promote acute myocarditis onset, potentially reducing female susceptibility [21]. However, once infection occurs, female mice are usually more severely infected than male mice, and estradiol demonstrates pro-arrhythmic effects in long QT syndrome type 2 (LQTS2) [22]. Collectively, these hormonal influences likely contribute to both the more severe disease manifestations and higher MT susceptibility observed in females. MT patients demonstrated only mild CRP and ESR abnormalities, significantly lower levels than NMA patients. The other indicators related to inflammation also exhibited downward trends, contrasting with elevated IL-10 levels. As an anti-inflammatory cytokine, IL-10 critically limits immune responses to prevent host damage during infection [23]. This paradox suggests that excessive IL-10 represents a failed compensatory response to overwhelming inflammation, which may facilitate viral persistence and lead to poor outcomes [24]. When identifying immunosuppression in MT patients at admission, we must recognize its clinical significance rather than dismissing it as mild inflammatory response—further clinical laboratory studies remain essential to confirm this association.

Although clinicians typically associate hypertriglyceridemia with MACE risk, our data paradoxically suggest that elevated plasma triglycerides are associated with a reduced risk of ventricular tachycardia and ventricular fibrillation (VT/VF) in patients with FM. As the organ with the highest metabolic demand in the human body [25], the heart primarily relies on fatty acid metabolism to maintain an adequate supply of ATP, primarily fueled by fatty acid (FA) β-oxidation (70% cardiac ATP) and glucose utilization (30%) [26]. FA is supplied to the heart as either free fatty acid (FFA) bound to albumin or as FA released from TG contained in chylomicrons or VLDL [27]. Low serum triglyceride levels are independently associated with increased mortality and malignant arrhythmias (ventricular tachycardia/fibrillation) in patients with fulminant myocarditis (FM). This association likely reflects the critical role of lipid metabolism in myocardial energy homeostasis. In FM, systemic inflammation and cytokine storm disrupt glucose-lipid metabolic pathways [28], leading to myocardial energy starvation and arrhythmogenic substrate formation. Concurrently, low triglycerides may serve as a biomarker for advanced disease severity or malnutrition [29], both of which are prevalent in critically ill FM patients. These findings parallel observations in heart failure cohorts [28], where triglyceride depletion correlates with arrhythmic events and poor outcomes [30], underscoring the interdependence of metabolic resilience and cardiac stability.

The MB patients exhibited characteristic ECG changes indicating significant conduction system disturbances. These multi-faceted abnormalities strongly suggest impaired myocardial conduction velocity alongside increased repolarization dispersion, creating an electrophysiological substrate highly susceptible to atrioventricular (AV) block. Crucially, the observed bradycardia in this cohort is mechanistically explained by its high prevalence of third-degree AV block. While FM patients typically develop compensatory tachycardia from fever, increased metabolic demand, and reduced contractility, those with MB paradoxically exhibit heart rate slowing. Viral invasion and autoimmune mechanisms—where autoantibodies activate complement systems, causing cytotoxicity [31]—likely damage cardiac conduction tissue, particularly AV nodal cells. This injury explains the high incidence of advanced AV block. Previous studies unexpectedly observed lower-than-anticipated sinus tachycardia rates in FM cohorts [7], possibly masked by conduction system impairment. Our findings demonstrate that heart rate slowing in FM patients highly indicates early conduction system compromise, warranting heightened vigilance. Paradoxically normal or reduced heart rates should not be misinterpreted as clinical stability but, rather, signal the urgent need for temporary pacemaker implantation. The observed delayed intrinsicoid deflection in MB patients indicates intraventricular conduction delay, attributable to the inflammation-induced slowing of electrical propagation [32]. While non-specific, this finding signifies diffuse myocardial involvement [33] and can serve as an auxiliary indicator of myocardial injury severity. Additionally, the MB group exhibited a significant increase in the E/Em ratio. This indicates diastolic dysfunction, suggesting impaired LV relaxation or filling pressures in MB patients [34]. This diastolic dysfunction may stem from the disruption of the coordinated timing of cardiac filling by independent atrial and ventricular contractions inherent in third degree AV block. Such electromechanical dyssynchrony likely adversely affects ventricular filling dynamics, ultimately contributing to diastolic dysfunction and potentially leading to worse clinical outcomes. The marked decrease in DBP observed in our patients with MB is indicative of cardiogenic shock. This results from a severe reduction in cardiac output due to myocardial injury, which triggers compensatory neurohormonal activation and peripheral vasoconstriction. While this helps maintain SBP, the low cardiac output causes a disproportionate fall in DBP, signifying acute left ventricular failure and less coronary blood flow [35].

This study has several limitations. First, some of the diagnosis was based on CMR findings, without histological confirmation by EMB in all cases. Second, viral genome analysis was not routinely performed in this study because, despite being recommended in guidelines, it is seldom used in real-world clinical practice. This is due to the lack of evidence that its results can guide treatment decisions in the acute setting of myocarditis [36]. Finally, the retrospective, single-center design and modest sample size may introduce selection bias and limit generalizability. A potential limitation is the declining rate of echocardiographic follow-up. However, clinical records and telephone interviews indicated that most patients lost to follow-up had fully recovered and chose not to return for routine imaging—a common phenomenon in benign post-myocarditis courses. Consequently, if anything, the loss to follow-up may have biased our results toward the null, as patients with excellent recovery (and thus normal LVEF) were underrepresented in late imaging. The fact that we still observed no significant difference in LVEF recovery between groups despite this bias strengthens the reliability of our conclusion that malignant arrhythmias do not impair long-term ventricular remodeling.

## 5. Conclusions

This study identifies distinct predictive factors for malignant arrhythmias in FM, stratified by arrhythmia subtype. Through logistic regression and ROC curve validation, we demonstrate that reduced serum triglyceride levels is an independent predictor for MT, while bradycardia, delayed intrinsicoid deflection, low diastolic blood pressure, and elevated E/Em ratio predict MB: TG achieves superior predictive value for MT (AUC = 0.678, Figure 3); HR, delayed intrinsicoid deflection; and DBP and E/Em show optimal performance for MB prediction (AUC = 0.838, Figure 4). Critically, the occurrence of MA is associated with worsened short-term prognosis in FM patients, though no significant impact on long-term outcomes was observed. These findings enable early risk stratification:

Patients with hypolipidemia require intensified monitoring for MT. Patients with bradycardia, delayed intrinsicoid deflection, low diastolic blood pressure, and elevated E/Em ratio warrant vigilance for MB. We propose that preemptive intervention targeting these high-risk profiles—particularly through combined biomarker assessment—may improve acute-phase outcomes. Future multi-center studies should validate these predictive models and explore underlying mechanisms linking TG metabolism and arrhythmogenesis in FM.

## Figures and Tables

**Table 1:** Comparison of the occurrence of major adverse cardiovascular events within 3 months after discharge between the malignant arrhythmias and non-malignant arrhythmias group.

Group	Total, *n*	Event of Occurrence, (*n* %)	Event-Free, *n* (%)	χ^2^ Value	*p*-Value
NMA	183	9 (4.9)	174 (95.1)	5.708	0.017
MA	58	9 (15.5)	49 (84.5)		

**Table 2:** Inflammatory factors in patients admitted with non-malignant arrhythmias and malignant tachyarrhythmia.

Characteristics	NMA (183)	MT (22)	*p*
IL-1β, pg/mL	5.0 (5.0, 7.6)	5.0 (5.0, 9.2)	0.714
IL-2R, U/mL	634 (412, 1133)	578 (425, 1240)	0.701
IL-6, pg/mL	15.4 (4.7, 76.7)	34.0 (10.0, 129.0)	0.209
IL-8, pg/mL	24.7 (10.0, 90.0)	20.7 (10.4, 72.3)	0.827
IL-10, pg/mL	5.0 (5.0, 18.9)	13.5 (5.0, 101.9)	0.037
TNF-α, pg/mL	12.7 (8.5, 21.4)	145 (6.7, 22.2)	0.797

Values are median (interquartile range) or *n* (%). IL, interleukin; IL-2R, interleukin-2 receptor; TNF, tumor necrosis factor.

**Table 3:** In-hospital management in patients admitted with non-malignant arrhythmias and malignant tachyarrhythmia.

Characteristics	NMA (183)	MT (22)	*p*
Temporary MCS devices			
IABP	159 (86.9)	21 (95.5)	0.415
ECMO	45 (24.6)	14 (63.6)	0.000
Pacemaker	25 (13.7)	11 (50.0)	0.000
Other support devices			
CRRT	40 (21.9)	10 (405.5)	0.015
IMV	26 (14.2)	8 (36.4)	0.019
CPR/Defibrillation	7 (3.8)	18 (81.8)	0.000
Vasoactive agent			
Dopamine	77 (42.5)	16 (72.7)	0.007
Metaraminol	19 (10.5)	8 (36.4)	0.002
Norepinephrine	19 (9.4)	1 (4.5)	0.720
Immunoregulatory therapy			
Initial dose of immunoglobulin, g	20 (10, 20)	15 (10, 20)	0.780
Total dose of immunoglobulin, g	60 (40, 90)	65 (50, 95)	0.146
Initial dose of glucocorticoids, mg	200 (200, 200)	200 (200, 400)	0.039
Total dose of glucocorticoids, mg	1000 (640, 1360)	1140 (700, 1890)	0.148
Other drugs therapy			
Antiviral drugs	180 (99.4)	22 (100)	1.000
ACEI/ARB	106 (57.9)	11 (50.0)	0.478
Beta blockers	113 (61.7)	15 (68.2)	0.556

Values are median (interquartile range) or *n* (%). NMA, malignant arrhythmia; MT, malignant tachyarrhythmia; MCS, mechanical circulation support; IABP, intra-aortic ballon pump; ECMO, extracorporeal membrane oxygenation; CRRT, continuous renal replacement therapy; IMV, invasive mechanical ventilation; CPR, cardiopulmonary resuscitation; ACEI, angiotensin-converting enzyme inhibitors; ARB, angiotensin II receptor blocker.

**Table 4:** Univariate regression analysis for malignant tachyarrhythmia characteristics in fulminant myocarditis.

Characteristics	OR	95% CI of OR		*p*
		Lower	Upper	
Male	0.255	0.090	0.721	0.010
LOS, per 1 day	1.024	0.977	1.073	0.332
SBP, per 10 mmHg	0.767	0.608	0.968	0.025
DBP, per 10 mmHg	0.644	0.471	0.881	0.006
P wave width, per 10 ms	0.792	0.627	1.000	0.050
TpTe interval, per 10 ms	1.118	1.011	1.236	0.030
Delayed intrinsicoid deflection	2.782	1.035	7.477	0.043
Average heart rate, per 10 beats	0.964	0.789	1.179	0.724
Total number of ventricular premature beats, per 10 beats	1.002	0.999	1.004	0.132
rMSSD	0.981	0.960	1.001	0.066
Pnn50	0.978	0.933	1.025	0.347
Left atrial enlargement	0.160	0.021	1.224	0.077
GLS, 1%	1.115	1.010	1.229	0.030
cTnI, per 1000 pg/mL	1.019	1.000	1.039	0.052
Lactate, per 1 mmol/L	1.236	1.093	1.397	0.001
ALT, per 100 U/L	1.043	1.004	1.084	0.029
AST, per 100 U/L	1.032	1.005	1.061	0.022
D-Dimer, per mg/L	1.083	1.009	1.161	0.026
PT, per s	1.023	0.994	1.052	0.117
LDH, per 10 U/L	0.999	0.997	1.000	0.158
Hemoglobin, per g/L	0.984	0.966	1.003	0.099
Triglyceride, per mmol/L	0.347	0.133	0.906	0.031
CRP, per 10 mg/L	0.929	0.846	1.016	0.104
ESR, per 10 mm/H	0.838	0.632	1.110	0.218
Calcium, per mmol/L	1.060	0.943	1.192	0.332
Corrected Calcium, per mmol/L	0.970	0.853	1.103	0.643
Magnesium, per mmol/L	0.809	0.595	1.101	0.177
Phosphorus, per mmol/L	2.492	1.235	5.028	0.011
IL-10, per pg/mL	1.001	0.999	1.004	0.181
Pacemaker	6.320	2.478	16.117	0.000
ECMO	5.367	2.114	13.623	0.000
CRRT	2.979	1.200	7.397	0.019
IMV	3.451	1.318	9.036	0.012
Initial dose of glucocorticoids, per 100 mg	1.607	1.031	2.506	0.036
Dopamine	3.602	1.347	9.630	0.011
Metaraminol	4.872	1.810	13.115	0.002

OR, odds ratio; CI, confidence interval; LOS, length of stay; SBP, systolic blood pressure; DBP, diastolic blood pressure; rMSSD, root mean square of successive differences; Pnn50, percentage of successive RR intervals that differ by more than 50 ms; GLS, global longitudinal strain; cTnI, cardiac troponin I; ALT, alanine aminotransferase; AST, aspartate aminotransferase; PT, prothrombin time; LDH, lactate dehydrogenase; CRP, C-reactive protein; ESR, erythrocyte sedimentation rate; IL-10, interleukin-10; ECMO, extracorporeal membrane oxygenation; CRRT, continuous renal replacement therapy; IMV, invasive mechanical ventilation.

**Table 5:** VIF results for variables.

Characteristics	Tolerance	VIF
Male	0.795	1.257
SBP, per 10 mmHg	0.580	1.724
DBP, per 10 mmHg	0.551	1.807
TpTe interval, per 10 ms	0.851	1.175
Delayed intrinsicoid deflection	0.805	1.243
GLS, 1%	0.703	1.422
cTnI, per 1000 pg/mL	0.752	1.330
Lactate, per 1 mmol/L	0.472	2.117
ALT, per 100 U/L	0.126	7.936
AST, per 100 U/L	0.121	8.233
D-Dimer, per mg/L	0.672	1.489
Triglyceride, per mmol/L	0.875	1.143
Phosphorus, per mmol/L	0.620	1.612
ECMO	0.504	1.984
Pacemaker	0.748	1.336
CRRT	0.640	1.564
IMV	0.653	1.532
Initial dose of glucocorticoids, per 100 mg	0.682	1.467
Dopamine	0.679	1.473
Metaraminol	0.694	1.440

VIF, variance inflation factor; SBP, systolic blood pressure; DBP, diastolic blood pressure; GLS, global longitudinal strain; cTnI, cardiac troponin I; ALT, alanine aminotransferase; AST, aspartate aminotransferase; ECMO, extracorporeal membrane oxygenation; CRRT, continuous renal replacement therapy; IMV, invasive mechanical ventilation.

**Table 6:** Multivariate regression analysis for malignant tachyarrhythmia characteristics in fulminant myocarditis.

Characteristics	OR	95% CI of OR		*p*
		Lower	Upper	
Male	0.231	0.039	1.353	0.104
SBP, per 10 mmHg	0.840	0.573	1.231	0.370
DBP, per 10 mmHg	0.997	0.545	1.824	0.993
TpTe interval, 10 ms	1.145	0.957	1.369	0.139
Delayed intrinsicoid deflection	2.669	0.534	13.330	0.232
GLS, 1%	0.987	0.821	1.187	0.892
cTnI, per 1000 pg/mL	1.030	0.990	1.071	0.141
Lactate, per 1 mmol/L	1.039	0.786	1.374	0.787
AST, per 100 U/L	0.999	0.994	1.005	0.803
D-Dimer, per mg/L	1.113	0.965	1.285	0.141
Triglyceride, per mmol/L	0.121	0.017	0.843	0.033
Phosphorus, per mmol/L	1.669	0.539	5.168	0.375
Initial dose of glucocorticoids, per 100 mg	0.546	0.193	1.543	0.253
Pacemaker	4.012	0.899	17.900	0.069
CRRT	1.797	0.359	9.001	0.476
IMV	0.506	0.083	3.092	0.506
ECMO	1.684	0.236	11.995	0.603
Dopamine	1.506	0.316	7.184	0.607
Metaraminol	4.618	0.658	32.417	0.124

OR, odds ratio; CI, confidence interval; SBP, systolic blood pressure; DBP, diastolic blood pressure; GLS, global longitudinal strain; cTnI, cardiac troponin I; AST, aspartate aminotransferase; ECMO, extracorporeal membrane oxygenation; CRRT, continuous renal replacement therapy; IMV, invasive mechanical ventilation.

## Data Availability

The data presented in this study are available on request from the corresponding author due to ethical restrictions.

## Supplementary Materials

The following supporting information can be found online at: https://www.mdpi.com/article/10.3390/jcdd13010014/s1, Table S1: Patient Completion Rates of Echocardiographic Examination at Each Follow-up Time Point in 3 Years; Table S2: Comparison of the occurrence of major adverse cardiovascular events over 3 months after discharge between the malignant arrhythmias and non-malignant arrhythmias group; Table S3: Clinical characteristics in patients admitted with non-malignant arrhythmias and malignant tachyarrhythmia; Table S4: Clinical characteristics in patients admitted with non-malignant arrhythmias and malignant bradyarrhythmia group; Table S5: Inflammatory factors in patients admitted with non-malignant arrhythmias and malignant bradyarrhythmia group; Table S6: In-hospital management in patients admitted with non-malignant arrhythmias and malignant bradyarrhythmia group; Table S7: Univariate regression analysis for malignant bradyarrhythmia characteristics in fulminant myocarditis; Table S8: VIF results for variables; Table S9; Multivariate regression analysis for malignant bradyarrhythmia characteristics in fulminant myocarditis.

## Abbreviations

The following abbreviations are used in this manuscript:

FM	Fulminant Myocarditis
MA	Malignant Arrhythmia
MB	Malignant Bradyarrhythmia
MT	Malignant Tachyarrhythmia
NMA	Non-Malignant Arrhythmia
