# Supplementary material for: Evaluation of Prognosis and Risk Factors of Fulminant Myocarditis Complicated with Malignant Arrhythmia
Source: J Cardiovasc Dev Dis. 2025 Dec 24;13(1):14. doi: 10.3390/jcdd13010014 (PMC12842383; doi:10.3390/jcdd13010014)
Supplement: Supplementary file 1 [file jcdd-13-00014-s001.zip › jcdd-3991892-supplementary.pdf]

Table S1. Patient Completion Rates of Echocardiographic Examination at Each Follow-up Time Point in 3 Years

Time Point	Total Patients	Echo Available	Completion Rate (%)
Baseline	241	241	100
1 month	241	138	57.3
3 month	226	101	44.7
6 month	204	106	52.0
12 month	189	90	47.6
18 month	171	36	21.1
24 month	150	54	36.0
30 month	128	13	10.2
36 month	107	27	25.2

Table S2. Comparison of the occurrence of major adverse cardiovascular events over 3 months after discharge between the malignant arrhythmias and non-malignant arrhythmias group

Group	total, n	event of occurrence, (n %)	event-free, n (%)	χ^2 Value	P value
NMA	183	4（2.2）	179（97.8）	0.003	0

MA	58	2（3.4）	56（96.6）		

Table S3. Clinical characteristics in patients admitted with non-malignant arrhythmias and malignant tachyarrhythmia.

Characteristics	NMA (183)	MT (22)	P
Male	98（53.6）	5（22.7）	0.006
Age, y	36（22,49）	34（23,46）	0.936
TFOHA, d	3（2,5）	3（2,4）	0.208
LOS, d	10（8,14）	14（10,19）	0.024
SBP, mmHg	101（90,112）	94（81,104）	0.020
DBP, mmHg	65±13	56±16	0.004
Smoking status			0.359
Never	147 (80.3)	20 (90.9)	
Former or Current	36（19.7）	2（9.1）	
Drinking status			0.254
Never	165 (90.2)	22(100)	
Former or Current	18（9.8）	0	
ECG at admission			
ST-T segment abnormalities	129（70.5）	18（81.8）	0.265
HR, per minute	93（80,117）	97（67,116）	0.536
P wave width, ms	92（76,100）	82（60,93）	0.035
QRS wave width, ms	96（84,118）	95（74,134）	0.633
PR interval, ms	150（136,169）	151（118,171）	0.527
RV5+SV1, mV	0.91（0.48,1.56）	0.54（0.27,1.57）	0.107

QTc prolongation	82（45.3）	11（50.0）	0.676
Frontal QRS-T angle,°	28.0（-21.9,76.0）	19.7（-78.4,105.7）	0.837
TpTe interval, ms	81（73,117）	97（78,150）	0.030
Delayed intrinsicoid deflection	26（14.4）	7（31.8）	0.074
Delayed QRS transition	63（34.8）	7（31.8）	0.781
Dynamic Electrocardiogram at admission			
Average heart rate	80（70,93）	74（69,85）	0.059
Maximum heart rate	115±18	113±19	0.759
Minimum heart rate	57（50,70）	55（49,66）	0.550
Total number of supraventricular premature beats	16（3,131）	16（2,141）	0.740
Total number of ventricular premature beats	14（1,92）	103（7,1083）	0.019
SDNN	80（51,106）	87（65,119）	0.288
SDANN	65（41,94）	64（47,102）	0.526
rMSSD	28（18,56）	43（29,79）	0.031
Pnn50	5（1,16）	11（8,17）	0.079
Triangular Index	19.9±10.5	19.0±11.1	0.732
Echocardiography at admission			
LVEF, %	40（28,52）	33（25,44）	0.120
Left atrial enlargement	42（23.0）	1（4.5）	0.084
Left ventricle dilation	23（12.6）	2（9.1）	0.900
E/A	1.5（1.0,2.3）	1.6（1.3,2.2）	0.424
GLS, %	-9.7±5.0	-7.3±4.3	0.027

E/Em	12（9,18）	13（7,16）	0.673
Admission laboratory tests			
cTnI, pg/mL	17387（3818,42762）	41931（16914,5000）	0.023
NT-proBNP, pg/mL	4914（1744,10676）	4891（2001,10116）	0.998
Lactate, mmol/L	2.2（1.5,3.4）	3.2（1.8,7.3）	0.041
D-Dimer, mg/L	1.7（0.7,3.6）	3.5（1.4,8.0）	0.008
APTT, s	42.7（36.8,64.0）	49.0（34.9,95.9）	0.417
PT, s	14.8（14.0,16.2）	15.9（14.3,19.8）	0.040
WBC, 10^9/L	9.5（7.0,13.3）	9.0（7.2,13.8）	0.970
Neutrophil, 10^9/L	7.5（5.3,11.1）	7.0（6.0,12.5）	0.692
Lymphocyte, 10^9/L	1.0（0.7,1.7）	0.9（0.6,1.1）	0.146
NLR	7.3（3.8,13.8）	8.9（5.5,18.3）	0.218
Hemoglobin, g/L	126（112,140）	118（103,130）	0.046
Platelet, 10^9/L	198（148,243）	165（146,233）	0.303
Triglyceride, mmol/L	1.1（0.7,1.5）	0.9（0.6,1.1）	0.034
HDL, mmol/L	0.9（0.6,1.1）	0.9（0.6,1.0）	0.938
LDL, mmol/L	2.0（1.4,2.6）	1.9（1.4,2.5）	0.585
ALT, U/L	49（28,90）	90（43,227）	0.038
AST, U/L	122（61,260）	165（117,407）	0.018
Scr, umol/L	77（61,102）	86（72,134）	0.140
LDH, U/L	448（320,719）	538（409,1188）	0.093
CRP, mg/L	32.6（9.4,79.0）	11.5（4.5,26.4）	0.021
ESR, mm/H	12（5,24）	7（3,17）	0.033
Potassium, mmol/L	4.1（3.8,4.4）	4.2（3.8,4.5）	0.335
Sodium, mmol/L	137（134,140）	136（134,142）	0.933

Chloride, mmol/L	101（98,104)	100（95,105）	0.404
Calcium, mmol/L	2.1（2.0,2.2)	2.0（1.8,2.1）	0.005
Corrected Calcium, mmol/L	2.2（2.1,2.3)	2.1（2.0,2.2）	0.006
Magnesium, mmol/L	0.9（0.8,1.0)	01.0（0.8,1.4）	0.084
Phosphorus, mmol/L	1.0（0.8,1.3)	1.3（0.9,1.6）	0.035
CMR	143（78.1%)	16（72.7%)	0.761
EMB	79（43.2%)	11（50.0%)	0.689
CMR/EMB	169（92.3%)	20（90.9%)	1.000

Values are mean ± standard deviation, median (interquartile range) or n (%). NMA, malignant arrhythmia; MT, malignant tachyarrhythmia; TFOHA, time from onset to hospital admission; LOS, length of stay; SBP, systolic blood pressure; DBP, diastolic blood pressure; ECG, electrocardiogram; HR, heart rate; SDNN, standard deviation of normal-to-normal intervals; SDANN, standard deviation of averages of normal-to-normal intervals; rMSSD, root mean square of successive differences; Pnn50, percentage of successive RR intervals that differ by more than 50 ms; LVEF, left ventricular ejection fraction; E/A, Early to late diastolic velocity ratio; GLS, global longitudinal strain; E/Em, E wave/peak early diastolic velocity of the mitral annulus; cTnI, cardiac troponin I; NT-proBNP, N-terminal pro-B-type natriuretic peptide; APTT, activated partial thromboplastin time; PT, prothrombin time; WBC, white blood cell; NLR, neutrophil-to-lymphocyte ratio; HDL, high-density lipoprotein; LDL, low-density lipoprotein; ALT, alanine aminotransferase; AST, aspartate aminotransferase; Scr, serum creatinine; LDH, lactate dehydrogenase; CRP, C-reactive protein; ESR, erythrocyte sedimentation rate; CMR, cardiac magnetic resonance; EMB, endocardial myocardial biopsy.

Table S4. Clinical characteristics in patients admitted with non-malignant arrhythmias and malignant bradyarrhythmia group

Characteristics	NMA (183)	MB (36)	P
Male	98（53.6)	20（55.6)	0.826
Age, y	36（22,49)	47（27,57)	0.026
TFOHA, d	3（2,5)	3（2,5)	0.817

LOS, d	10（8,14）	12（7,16）	0.625
SBP, mmHg	101（90,112）	95（83,112）	0.123
DBP, mmHg	65±13	58±11	0.004
Smoking status			0.534
Never	141（77.0）	26（72.2）	
Former or Current	42（23.0）	10（27.8）	
Drinking status			0.669
Never	165（90.2）	31（86.1）	
Former or Current	18（9.8）	5（13.9）	
ECG at admission			
ST-T segment abnormalities	122（66.7）	27（75.0）	0.327
HR, per minute	93（79,117）	81（60,98）	0.001
P wave width, ms	92（80,100）	80（67,94）	0.002
QRS wave width, ms	96（84,116）	130（98,153）	<0.001
PR interval, ms	150（133,169）	166（149,192）	0.004
RV5+SV1, mV	0.94（0.48,1.59）	0.76（0.24,1.21）	0.012
QTc prolongation	80（43.7）	19（52.8）	0.318
Frontal QRS-T angle,°	22.4（-36.6,60.5）	-36.5（-118.4,55.6）	0.011
TpTe interval, ms	81（71,116）	98（70,124）	0.195
Delayed intrinsicoid deflection	24（13.1）	18（50.0）	<0.001
Delayed QRS transition	62（33.9）	21（58.3）	0.006
Dynamic Electrocardiogram at admission			
Average heart rate	80（69,93）	79（63,89）	0.298
Maximum heart rate	115±18	105±19	0.005
Minimum heart rate	57（50,70）	60（50,64）	0.767
Total number of supraventricular premature beats	15（1,164）	31（4,254）	0.315
Total number of ventricular premature beats	15（2,100）	33（4,241）	0.405
SDNN	79（51,106）	86（51,112）	0.968
SDANN	65（41,93）	66（35,89）	0.564
rMSSD	30（18,56）	39（13,79）	0.279
Pnn50	5（1,17）	9（0,27）	0.295
Triangular Index	17.5（11.5,25.7）	14.3（5.8,23.4）	0.043
Echocardiography at admission			
LVEF, %	40（28,52）	40（32,52）	0.732
Left atrial enlargement	44（24.0）	9（25.0）	0.903
Left ventricle dilation	25（13.7）	3（8.3）	0.547
E/A	1.6±0.9	1.7±0.8	0.488
GLS, %	-9.4±5.1	-8.4±5.0	0.372
E/Em	13（9,19）	19（12,27）	<0.001

Admission laboratory tests			
cTnI, pg/mL	17387 (3818,42762)	19591 (6497,47187)	0.612
NT-proBNP, pg/mL	5086 (1768,10826)	6449 (1972,18446)	0.142
Lactate, mmol/L	2.3 (1.5,3.6)	3.0 (1.7,4.7)	0.056
D-Dimer, mg/L	1.7 (0.7,3.6)	2.0 (1.1,4.8)	0.166
APTT, s	42.6 (36.8,61.6)	42.8 (35.5,98.2)	0.828
PT, s	14.9 (14.0,16.4)	15.5 (14.0,18.1)	0.332
WBC, 10^9/L	9.5 (7.0,13.3)	9.8 (7.0,12.4)	0.931
Neutrophil, 10^9/L	7.5 (5.3,11.1)	7.5 (5.3,11.1)	0.815
Lymphocyte, 10^9/L	1.0 (0.7,1.7)	1.0 (0.6,1.5)	0.614
NLR	7.3 (3.8,13.8)	9.4 (4.8,13.2)	0.434
Hemoglobin, g/L	126 (112,140)	126 (111,138)	0.904
Platelet, 10^9/L	198 (148,243)	189 (148,243)	0.691
Triglyceride, mmol/L	1.1 (0.7,1.9)	1.1 (0.7,1.5)	0.493
HDL, mmol/L	0.8 (0.6,1.1)	0.8 (0.6,1.0)	0.915
LDL, mmol/L	1.8 (1.3,2.5)	1.8 (1.4,2.5)	0.813
ALT, U/L	49 (28,93)	87 (32,257)	0.039
AST, U/L	121 (61,243)	145 (79,483)	0.145
Scr, umol/L	77 (61,102)	73 (59,116)	0.772
LDH, U/L	448 (305,719)	559 (367,1283)	0.077
CRP, mg/L	31.8 (9.0,79.0)	23.1 (8.0,63.9)	0.360
ESR, mm/H	12 (5,24)	7 (4,18)	0.215
Potassium, mmol/L	4.1 (3.8,4.4)	4.2 (3.9,4.6)	0.128
Sodium, mmol/L	137 (134,140)	137 (132,139)	0.274
Chloride, mmol/L	101 (98,104)	101 (98,104)	0.548
Calcium, mmol/L	2.1 (2.0,2.2)	2.0 (2.0,2.2)	0.141
Corrected Calcium, mmol/L	2.2 (2.1,2.3)	2.2 (2.0,2.3)	0.245
Magnesium, mmol/L	0.9 (0.8,1.0)	0.9 (0.8,1.0)	0.692
Phosphorus, mmol/L	1.0 (0.8,1.3)	1.1 (0.9,1.4)	0.079
CMR	143 (78.1%)	27 (75%)	0.679
EMB	79 (43.2%)	18 (50%)	0.451
CMR/EMB	169 (92.3%)	33 (91.7%)	1.000

Values are mean±standard deviation, median (interquartile range) or n (%). TFOHA, time from onset to hospital admission; LOS, length of stay; SBP, systolic blood pressure; DBP, diastolic blood pressure; ECG, electrocardiogram; HR, heart rate; SDNN, standard deviation of normal-to-normal intervals; SDANN, standard deviation of averages of normal-to-normal intervals; rMSSD, root mean square of successive differences; Pnn50, percentage of successive RR intervals that differ by more than 50 ms; LVEF, left ventricular ejection fraction; E/A, Early to late diastolic velocity ratio; GLS, global longitudinal strain; E/Em, E wave/peak early diastolic velocity of the

mitral annulus; cTnI, cardiac troponin I; NT-proBNP, N-terminal pro-B-type natriuretic peptide; APTT, activated partial thromboplastin time; PT, prothrombin time; WBC, white blood cell; NLR, neutrophil-to-lymphocyte ratio; HDL, high-density lipoprotein; LDL, low-density lipoprotein; ALT, alanine aminotransferase; AST, aspartate aminotransferase; Scr, serum creatinine; LDH, lactate dehydrogenase; CRP, C-reactive protein; ESR, erythrocyte sedimentation rate; CMR, cardiac magnetic resonance; EMB, endocardial myocardial biopsy.

Table S5. Inflammatory factors in patients admitted with non-malignant arrhythmias and malignant bradyarrhythmia group

Characteristics	NMA (183)	MB (36)	P
IL-1β, pg/mL	5.0（5.0,8.1）	5.0（5.0,5.3）	0.272
IL-2R, U/mL	611（400,999）	684（471,925）	0.613
IL-6, pg/mL	15.5（4.7,76.7）	15.1（5.0,65.7）	0.697
IL-8, pg/mL	24.7（10.0,90.0）	210.（8.4,108.7）	0.806
IL-10, pg/mL	5.0（5.0,18.4）	5.0（5.0,10.6）	0.829
TNF-α,, pg/mL	12.（8.4,20.7）	13.2（10.0,16.7）	0.501

Values are median (interquartile range) or n (%). IL, interleukin; IL-2R, interleukin-2 receptor; TNF, tumor necrosis factor.

Table S6. In-hospital management in patients admitted with non-malignant arrhythmias and malignant bradyarrhythmia group

Characteristics	NMA (183)	MB (36)	P
Temporary MCS devices			
IABP	149（81.4）	28（77.8）	0.612
ECMO	61（33.3）	13（36.1）	0.747
Pacemaker	29（15.8）	27（75.0）	<0.001
Other support devices			
CRRT	40（21.9）	12（33.3）	0.139
IMV	34（18.6）	6（16.7）	0.786
CPR/Defibrillation	16（8.7）	14（38.9）	<0.001
Vasoactive agent			

Dopamine	77 (42.1)	20 (55.6)	0.137
Metaraminol	19 (10.4)	4 (11.1)	1.000
Norepinephrine	17 (9.3)	2 (5.6)	0.686
Immunoregulatory therapy			
Initial dose of immunoglobulin	20 (10,20)	15 (10,20)	0.884
Total dose of immunoglobulin	60 (40,90)	50 (36,80)	0.350
Initial dose of glucocorticoids	200 (200,200)	200 (200,200)	0.930
Total dose of glucocorticoids	1000 (640,1360)	880 (610,1435)	0.575
Other drugs therapy			
Antiviral drugs	182 (99.5)	3 (94.4)	0.114
ACEI/ARB	111 (60.7)	1 (41.7)	0.035
Beta blockers	113 (61.7)	15 (41.7)	0.025

Values are median (interquartile range) or n (%). MCS, mechanical circulation support; IABP, intra-aortic ballon pump; ECMO, extracorporeal membrane oxygenation; CRRT, continuous renal replacement therapy; IMV, invasive mechanical ventilation; CPR, cardiopulmonary resuscitation; ACEI, angiotensin-converting enzyme inhibitors; ARB, angiotensin II receptor blocker.

Table S7. Univariate regression analysis for malignant bradyarrhythmia characteristics in fulminant myocarditis.

Characteristics	OR	95% CI of OR		P
		Lower	Upper	
Age, per y	1.025	1.003	1.048	0.029
DBP, per 10mmHg	0.662	0.496	0.882	0.005
HR, per 10beats	0.752	0.640	0.884	0.001
P wave width, per 10ms	0.740	0.612	0.896	0.002
PR interval, per 10ms	1.131	1.033	1.239	0.008
QRS wave width, 10ms	1.261	1.129	1.424	<0.001

RV5+SV1, mV	0.553	0.321	0.951	0.032
Frontal QRS-T angle, per 10°	0.941	0.902	0.981	0.005
Delayed intrinsicoid deflection	6.625	3.032	14.477	<0.001
Delayed QRS transition	2.732	1.317	5.669	0.007
Maximum heart rate, per 10beats	0.752	0.615	0.921	0.006
Triangular Index	0.964	0.964	1.001	0.056
E/Em	1.058	1.020	1.097	0.002
Lactate, per 1 mmol/L	1.092	0.941	1.266	0.248
ALT, per 100 U/L	1.049	1.002	1.097	0.039
LDH, per 10U/L	1.008	1.002	1.015	0.016
Phosphorus, per mmol/L	1.529	0.944	2.478	0.084

OR, odds ratio; CI, confidence interval; DBP, diastolic blood pressure; HR, heart rate; E/Em, E wave/peak early diastolic velocity of the mitral annulus; ALT, alanine aminotransferase; LDH, lactate dehydrogenase;

Table S8. VIF results for variables

Characteristics	Tolerance	VIF
Age, per y	0.767	1.309
DBP, per 10mmHg	0.926	1.080
HR, per 10beats	0.750	1.333
P wave width, per 10ms	0.881	1.135
PR interval, per 10ms	0.812	1.231
QRS wave width, 10ms	0.672	1.489
RV5+SV1, mV	0.762	1.312
Frontal QRS-T angle, per 10°	0.877	1.140
Delayed intrinsicoid deflection	0.672	1.489
Delayed QRS transition	0.714	1.400
Maximum heart rate, per 10beats	0.676	1.480
E/Em	0.889	1.125
ALT, per 100 U/L	0.539	1.855
LDH, per 10U/L	0.446	2.242
cTnI, per 1000pg/mL	0.757	1.320

VIF, variance inflation factor; DBP, diastolic blood pressure; HR, heart rate; E/Em, E wave/peak early diastolic velocity of the mitral annulus; ALT, alanine aminotransferase; LDH, lactate dehydrogenase; cTnI, cardiac troponin I.

Table S9. Multivariate regression analysis for malignant bradyarrhythmia characteristics in fulminant myocarditis.

Characteristics	OR	95% CI of OR		P
		Lower	Upper	
Age, per y	0.993	0.962	1.025	0.670
DBP, per 10mmHg	0.662	0.454	0.967	0.033
HR, per 10beats	0.787	0.642	0.965	0.022
P wave width, per 10ms	0.833	0.652	1.065	0.145
PR interval, per 10ms	1.050	0.938	1.175	0.398
QRS wave width, 10ms	1.134	0.990	1.300	0.070
RV5+SV1, mV	0.750	0.374	1.506	0.419
Frontal QRS-T angle, per 10°	0.964	0.916	1.015	0.163
Delayed intrinsicoid deflection	3.392	1.090	10.559	0.035
Delayed QRS transition	0.751	0.250	2.256	0.610
Maximum heart rate, per 10beats	1.031	0.789	1.348	0.823
E/Em	1.061	1.012	1.113	0.014
ALT, per 100 U/L	1.006	0.909	1.114	0.904
LDH, per 10U/L	1.008	0.994	1.023	0.276
cTnI, per 1000pg/mL	0.973	0.942	1.005	0.100

OR, odds ratio; CI, confidence interval; DBP, diastolic blood pressure; HR, heart rate; E/Em, E wave/peak early diastolic velocity of the mitral annulus; ALT, alanine aminotransferase; LDH, lactate dehydrogenase; cTnI, cardiac troponin I.
